# Supplementary material for: Are carbon emissions trading and green financial instruments synergistic? -Comprehensive quantitative research based on content analysis
Source: PLoS One. 2024 Mar 7;19(3):e0298601. doi: 10.1371/journal.pone.0298601 (PMC10919601; doi:10.1371/journal.pone.0298601)
Supplement: S1 Data — (ZIP) [file pone.0298601.s001.zip › Data/地级市Z_F工作报告.zip!/║■▒▒/╧σ╤⌠╩╨/└┤╫╘╣1⁄2╓┌║┼í╛┬φ┐╦╩2╛▌═°í┐_2017.docx]

各位代表：

　　现在，我代表市人民政府向大会作工作报告，请予审议，并请政协委员和其他列席人员提出意见。

　　一、过去五年工作的回顾

　　过去的五年，全市上下在市委的坚强领导下，主动适应新常态，积极应对经济下行的困难局面，持续推进“两个中心、四个襄阳”建设，全市经济社会发展实现历史性跨越。

　　——综合实力大幅攀升。地区生产总值先后迈上2000亿元、3000亿元台阶，预计2016年地区生产总值达到3650亿元，经济总量由全国城市第75位跃升至第56位。规模以上工业总产值实现倍增，地方公共财政预算收入按可比口径实现翻两番，累计完成全社会固定资产投资、招商实际投资、工业投资分别是前五年的4倍、4.9倍、3.7倍，集聚世界500强企业28家。高新区综合实力居国家级高新区第33位，6个县（市）和襄州区在全省县域经济分类考核中均列第一方阵，枣阳市挺进全国县域经济与县域基本竞争力百强。全市主要经济指标在全省的占比明显提高，在汉江流域的经济首位度显著提升。

　　——产业转型明显加快。万亿工业强市建设扎实推进，汽车产业和农产品加工业产值分别突破2000亿元，装备制造业产值突破1000亿元，我市成为新能源汽车、军民结合、再生资源利用三大国家新型工业化产业示范基地。现代服务业突破性发展，“襄阳云谷”成为全省信息化支撑平台，我市成为国家二级物流园区布局城市、电子商务示范创建试点。农业发展方式转变加快，“中国有机谷”上升为省级战略，整市进入国家现代农业示范区行列。

　　——城乡面貌深刻变化。中心城市能级蝶变，东津新区骨架拉开，旧城更新成效显著，中心城区路网体系初步建立，“一心四城”的空间格局基本形成，荣膺国家森林城市、国家卫生城市。“四位一体”的城镇体系初步建立，襄宜南一体化、河谷组群、枣阳副中心、保康生态旅游试验区建设取得积极进展，“四化同步”示范镇和美丽乡村建设成效显著。区域性综合交通枢纽地位显著增强，铁路枢纽、国家公路主枢纽和航空港吞吐能力明显增强，实现了县县通高速。生态环境持续改善，节能减排任务全面完成，我市成为全国水生态文明试点城市和国家可持续发展实验区。

　　——改革创新持续突破。实施100多项全国、全省改革创新试点，行政管理体制改革、商事制度改革、农村土地制度改革、户籍制度改革、供给侧结构性改革走在全省前列。开放发展水平显著提高，湖北自贸区襄阳片区获批建设，外贸出口额、外商直接投资额分别居全省第2位和第3位，国际友城发展到19个。创新驱动发展战略深入实施，我市成为全国科技进步示范市和国家创新型城市试点。

　　——民生福祉全面提升。民生投入力度不断加大，改善民生三年目标任务如期完成。统筹城乡的社会保障和救助体系全面建立，社会保障水平不断提高，就业规模不断扩大，城乡居民收入持续增长，33.7万贫困人口稳定脱贫，44万城乡群众的住房条件得到改善。公共服务水平显著提高，教育均衡发展、保障性住房建设等工作居全省前列，居民主要健康指标优于全国全省平均水平，我市成为国家公共文化服务体系示范区。社会治理不断加强，信访维稳力度不断加大，公共法律服务体系基本建立，我市荣获“全国综治优秀市”称号，成为国家安全发展和食品安全试点城市。

　　与此同时，国防后备力量建设不断加强，军政军民团结日益巩固，获得“全国双拥模范城”三连冠。全市其它各个领域、各条战线的工作都取得了显著成绩。

　　五年来，我们以深入开展党的群众路线教育实践活动、“三严三实”专题教育和“两学一做”学习教育为契机，不断加强政府自身建设，全面推进依法行政、从严治政，认真执行市人大及其常委会各项决议决定，自觉接受市人大的法律监督、工作监督和市政协的民主监督，认真办理人大代表议案、建议和政协建议案、提案，严格执行中央八项规定精神，持续加强党风廉政建设，法治政府、服务型政府和廉洁政府建设取得显著成效，政风行风明显改进，服务效能不断提升。

　　刚刚过去的2016年，我们牢固树立和落实新发展理念，以“补短板”为重点推进供给侧结构性改革，全市呈现“经济稳中向好、产业结构优化、发展质效提升、民生持续改善”的态势，实现了“十三五”良好开局。预计地区生产总值比上年增长8.5%，规模以上工业增加值增长10%，全社会固定资产投资增长18%，社会消费品零售总额增长12.5%，城镇和农村常住居民人均可支配收入分别增长9.8%和9.7%，计划生育、节能减排、安全生产、居民消费价格指数等约束性指标全面完成省定目标。

　　一年来，我们主要抓了五个方面的工作：

　　（一）强化经济发展支撑。全力推进招商引资。全年招商引资实际投资额2200亿元，工业项目投资占比超过70%，投资88亿元的猛狮科技新能源产业基地项目落户宜城。全力推进项目建设。实施省市重点项目130个，完成投资666.7亿元；竣工投产亿元以上工业项目200个，其中10亿元以上项目18个。全力服务实体经济。全面落实万亿工业强市建设措施，支持企业转型升级；开展降成本行动，为企业减负45亿元；推动金融改革创新，利用汉江产业基金和县域经济调度资金，帮助186家中小企业缓解了融资难；组织千名干部进千企，帮助解决了一大批实际问题。

　　（二）强力推进产业升级。加快推进工业转型升级。实施亿元以上技改项目172个，竣工投产85个，三环锻造、卫东化工成为国家智能制造试点示范企业；战略性新兴产业产值占规模以上工业的比重达到14.4%，“中国新能源汽车之都”建设取得重大突破，东风俊风和众泰两款纯电动轿车投产下线，骆驼动力电池一期项目竣工投产，雅致新能源乘用车、国能动力电池等项目开工建设。加快推进高端服务业发展。文化旅游、云服务、检测认证、电子商务、服务外包等新兴服务业发展提速，高新区成为全国服务外包示范基地；中心城区18个专业市场有序外迁，专业市场集聚区初具规模。加快推进现代农业建设。新增国家级和省级示范家庭农场39家，一二三产业融合发展得到国家农业部肯定，粮食生产再获丰收。加快推进创业创新。新认定高新技术企业148家，获得省级以上重大科技成果190项，中航精机荣获国家科学技术进步一等奖；襄阳科技城正式开园，全市新增高端人才创业创新团队23个、大学生创业者3123人，市场主体总量、增量均居全省第二位。

　　（三）统筹推进城乡建设。加强中心城市建设和管理。使用国开行、农发行政策性棚改贷款111.2亿元，改造棚户区房屋45299套，其中市区改造18869套，基本实现了货币化安置；东津新区成为首批国家产城融合示范区，东津高铁站城市综合配套工程开工建设，中心医院新院区投入使用；内环南线东延伸段、316和207国道市区段建成通车，绕城高速东段和南北轴线将于春节前通车，新增城市绿地307万平方米，开工建设海绵城市1121万平方米，城区污水实现全收集，城市管理水平进一步提高。加快建设重大基础设施。蒙华铁路、汉十高铁、郑万高铁建设快速推进，襄北编组站改扩建完工；麻竹高速襄阳西段、保宜高速襄阳段建成通车，建成一二级公路300公里、农村公路1000公里；襄阳机场航站楼主体完工，雅口航电枢纽开工建设，鄂北水资源配置工程建设进展顺利。积极推进美丽襄阳建设。空气质量优良天数比上年增加34天，汉江襄阳段水质稳定在Ⅱ类水平，22.8万农村居民饮水不安全问题得到有效解决，农村垃圾收集清运处理体系基本建立；自然保护区和湿地公园建设取得新的突破，“绿满襄阳”提升行动取得明显成效，新增省级绿色示范乡村121个、美丽宜居乡村建设试点28个。

　　（四）全面深化改革开放。持续推进简政放权，审批事项平均办结时间由38个工作日压缩至7个工作日，在全省率先推行电子营业执照、住所登记信息申报制和企业集群登记。充分发挥产业基金的撬动作用，积极推动政府和社会资本合作，吸引社会资本投入基础设施和实体经济467.8亿元；新增新三板和四板挂牌企业41家，上市公司股票增发融资34.1亿元，发行企业债券46.3亿元，争取国家专项建设基金21.4亿元。居住证制度正式实施，常住人口市民化进程加快，宜城新型城镇化综合试点和宅基地制度改革走在全国前列，集体林权“三权分置”改革经验在全国推广。保税物流中心通过国家验收，对“一带一路”沿线国家投资额同比增长33.1倍、出口额占全市出口总量的比重达到50%以上，襄阳轴承波兰项目成为全省重点支持的“一带一路”项目。

　　（五）持续加强民生保障。全市完成易地扶贫搬迁6130户，减少贫困人口11万，出列贫困村140个。培训各类劳动者13.5万人，新增城镇就业9.1万人。城乡社会保障水平进一步提高，全市统一的临时救助和医疗救助制度全面实施，“三无人员”自愿集中供养实现全覆盖，城镇公共租赁住房、租赁补贴和公积金保障覆盖面进一步扩大。学前教育实现新发展，全市县域义务教育均衡发展通过国家验收，普通高中教育质量保持全省领先。医药卫生改革和卫生计生事业发展取得新成效，我市成为全国城市公立医院综合改革试点城市、全国健康城市评价试点。各类惠民文化活动丰富多彩，全民阅读和“戏曲进校园”活动引起了主流媒体高度关注。城乡网格化管理进一步完善，创建全国综治“长安杯”取得积极成效，安全生产和食品药品安全监管有力，食品安全监管工作获得全国人大常委会执法检查组的高度评价。

　　各位代表！回顾过去，五年的历程充满艰辛，取得的成就令人振奋。襄阳五年来的变化和进步，得益于省委省政府和市委的坚强领导，得益于市人大、市政协的全力支持，得益于全市人民的共同奋斗。在此，我代表市人民政府，向全市人民，向各位人大代表、政协委员，向各民主党派、工商联、各人民团体和无党派人士，向驻襄人民解放军、武警官兵和公安干警，向所有投资者、创业者和建设者，向所有关心支持襄阳发展的同志们、朋友们，致以崇高的敬意和衷心的感谢！

　　各位代表！五年的成绩铸就了新的起点，但前进的道路上还有不少困难和挑战。我们必须看到：发展不够仍是襄阳最大的实际，实现经济总量占全省六分之一的目标任务十分艰巨；供给侧结构性矛盾突出，发展新动力支撑不足；经济下行压力持续加大，结构调整、动能转换带来的实体经济阵痛仍在加剧；改革进入深水区、调整触及深层次，化解矛盾的成本高、难度大；中心城市辐射带动能力不足，县域经济竞争力不强，统筹城乡发展的任务繁重；资源环境约束趋紧，环境承载压力日益加大，实现绿色发展显得尤为紧迫；基本公共服务供给不足，社会保障水平和城乡居民收入不高，与人民群众的期待相比还有较大差距，等等。我们一定以强烈的责任感和使命感，全力解决发展中的突出矛盾和问题，奋力开创改革发展新局面，决不辜负全市人民的重托和期望！

　　二、今后五年工作的总体安排

　　未来五年，是襄阳决胜率先全面小康、开启实现基本现代化新征程的关键时期。市十三次党代会科学研判走势、准确把握大势，赋予了襄阳未来发展新使命，这就是：提升首位度、打造增长极、争当排头兵，奋力开创“两个中心、四个襄阳”建设新局面。我们务必以坚定的政治定力、强烈的使命担当，抢抓机遇、乘势而上，敢为人先、奋发竞进，一步一个脚印地推动市委决策部署落地生根，不断开辟转型跨越新境界。

　　今后五年政府工作的指导思想是：高举中国特色社会主义伟大旗帜，以马克思列宁主义、毛泽东思想、邓小平理论、“三个代表”重要思想、科学发展观为指导，深入贯彻习近平总书记系列重要讲话精神和治国理政新理念新思想新战略，统筹推进“五位一体”总体布局和协调推进“四个全面”战略布局，引领经济发展新常态，牢固树立新发展理念，认真落实市十三次党代会部署，坚持创新驱动、智能转型，坚持生态优先、绿色发展，坚持改革引领、开放带动，坚持民生为本、共建共享，提升首位度、打造增长极、争当排头兵，奋力开创“两个中心、四个襄阳”建设的新局面，率先在全省全面建成小康社会，为开启基本实现现代化新征程打下坚实基础。

　　综合考虑，今后五年全市经济社会发展的主要目标是：主要经济指标增速高于全省平均水平、高于汉江流域平均水平、高于全国同类城市平均水平，发展质量和效益好于“十二五”时期，经济总量跻身全国城市50强，城镇和农村常住居民人均可支配收入与经济增长同步，全面完成精准脱贫、计划生育、安全生产、节能减排、居民消费价格指数等省定目标。

　　围绕实现上述目标，今后五年，必须始终坚持以经济建设为中心，用“第一力度”抓好“第一要务”，奋力推进各项工作走在全省和汉江流域前列。

　　（一）坚持创新发展，奋力推进转型升级走在全省和汉江流域前列

　　以供给侧结构性改革为主线，促进“中国制造2025襄阳行动”、“互联网+”和大众创业万众创新紧密结合，同步推进新动能培育与传统动能改造，增强竞争优势，壮大经济实力。

　　加快构建现代产业新体系。强力推进万亿工业强市建设。坚持一手抓支柱产业智能化改造，一手抓战略性新兴产业培育壮大，全面推进“一个龙头、六大支柱”产业转型升级，全力支持东风公司襄阳基地做大做强，着力打造“中国新能源汽车之都”，加快建设中西部地区重要的高端装备制造、电子信息、新能源新材料、大健康、军民融合等五大产业基地；力争到2021年，全市工业总产值突破1.4万亿元，汽车产业产值突破4000亿元、其中新能源汽车突破1000亿元，农产品加工业突破4000亿元，装备制造产业突破2000亿元，电子信息、纺织服装、医药化工产业分别突破1000亿元。强力推进现代服务业强市建设。加快壮大“襄阳云谷”，加快建设辐射中西部地区的检测认证中心、汉江流域金融服务中心和现代商贸物流中心，加快形成辐射汉江流域的生产性服务业、电子商务、文化旅游产业板块，着力打造中部地区重要的旅游集散地和旅游目的地，力争到2021年，社会消费品零售总额突破2280亿元，电子商务交易额突破1500亿元，现代物流业增加值突破1000亿元，服务业增加值占GDP的比重达到43%。强力推进现代农业强市建设。以建设高水平的国家现代农业示范区为抓手，加快构建现代农业生产体系、经营体系、产业体系，着力推进一二三产业融合发展；强化农业科技支撑，努力实现藏粮于地、藏粮于技，全市新建高标准农田100万亩，粮食总产量稳定在105亿斤以上，“三品一标”认证突破700个，力争把“中国有机谷”建成国家优质安全农产品生产示范区。

　　加快培育发展新动力。深入实施创新驱动发展战略，以建成国家创新型城市和创业型城市为目标，深入开展“双创”行动，培育发展“四新”经济。力争省级以上研发创新平台达到160个以上、众创空间和科技企业孵化器分别达到20个以上，各类市场主体总量达到55万户以上；高新技术企业突破600家，战略性新兴产业、高新技术产业增加值占GDP的比重分别达到14.6%、26%，积极创建全国质量强市示范城市和全国商标示范城市。实施人才强市战略，深入推进“隆中人才支持计划”，力争新引进100个国内外创新创业团队、200名科技领军人才、1000名高层次科技人才。聚焦转型升级抓招商、建项目，持续扩大有效投资，力争五年累计投资突破2.5万亿元。

　　加快构建发展新体制。全力推进供给侧结构性改革，扎实抓好“三去一降一补”。深入推进“放管服”改革，深化商事制度改革，持续优化营商环境。深入推进投融资体制改革，整合政府投融资平台，创新筹融资方式，壮大产业基金规模，推行政府和社会资本合作模式；加快形成资本市场的“襄阳板块”，力争上市公司达到15家，新三板、四板挂牌企业分别达到60家以上和600家以上。构建新型政商关系，加强各种所有制经济产权保护，大力发展民营经济和混合所有制经济。深入推进行政管理、公共服务、国企国资、农业农村、生态文明、财税等重点领域改革，激发经济社会发展的动力活力。

　　（二）坚持协调发展，奋力推进统筹城乡走在全省和汉江流域前列

　　坚持区域协同、城乡一体，全面提升城镇功能品位，着力形成具有襄阳特色的新型城镇化格局。

　　优化城市空间格局。启动新一轮城市总体规划编制工作，加快推进中心城区功能区建设，推动“一心四城”功能融合、协同发展，促进高新区和襄州区产城融合发展，加快建设东津新区、庞公新区、樊西新区，加大鱼梁洲城市“绿心”保护力度。统筹规划建设主城区周边乡镇，打造中心城区卫星城。加快推进襄宜南一体化，强化枣阳、河谷组群两个副中心的支撑作用，提升保康在鄂西生态文化旅游圈的地位。

　　推进城市功能跃升。全力打造全国重要的高铁和客货运综合枢纽，完善国家公路运输枢纽功能，加快建设汉江航运中心和区域性航空服务中心。完善市政功能体系，规划建设城市轨道交通，建成“两轴三环九放射”的骨干路网体系；加快建设“智慧城市”、“海绵城市”；统筹规划建设停车场、人行通道、公共厕所、消防站等公共设施，实施集中供暖、老旧小区电梯配套等民生工程，努力提升市民生活的便利度和舒适感。

　　全面提升城市品质。全力推进棚户区改造，推动城市功能再造、品质提升。建设山水园林城市，把好山好水好风光融入城市，完善城市生态体系，人均绿地面积达到12.8平方米。坚持以文塑城，促进具有襄阳特色的文化元素有机融入城市建设，加强历史文化街区、风景名胜区的保护利用，认真落实《襄阳古城墙保护条例》，按照“减人口、矮建筑、通城墙、连活水、弱交通”的要求，推进襄阳古城保护和开发，彰显历史文化名城特色魅力。

　　推动城乡协调发展。坚持城区经济和县域经济“两轮驱动”。全面落实市十三次党代会赋予各地的发展定位，高昂城区“龙头”，补强县域短板，到2021年，力争高新区综合实力跻身国家级高新区第一方阵，东津新区工业总产值突破1000亿元、枣阳冲刺2000亿元、襄州突破1500亿元，谷城、宜城、老河口突破1200亿元；支持襄城加快打造国内外有重要影响的文化名城、旅游“香城”，支持樊城加快创建国家级现代商贸物流示范区和区域性现代服务业中心，支持南漳建成国家有机农业和绿色发展示范县，支持保康建设全国生态旅游和文化小康示范县。推动城乡发展一体化。建立健全农业转移人口市民化机制，推动城镇常住人口基本公共服务全覆盖；加快建设美丽乡村，积极培育特色小镇，力争创建30个省级“四化同步”示范镇、300个省级宜居示范村。

　　（三）坚持绿色发展，奋力推进生态建设走在全省和汉江流域前列

　　牢固树立绿水青山就是金山银山的理念，筑牢全省重要的生态屏障，让良好生态环境成为人民生活质量的增长点，成功创建国家生态园林城市。

　　优化国土空间开发格局。强化重点生态功能区管控，落实国家主体功能区规划，统筹各类空间规划，推进“多规合一”，科学划定城市开发边界，推行绿色规划设计和建设，构建科学合理的生产空间、生活空间、生态空间格局。

　　推动绿色低碳循环发展。大力推广清洁能源，非化石能源占一次能源消费比重达到16%。积极发展绿色建筑、绿色设施、绿色交通，建设绿色出行设施，鼓励低碳产品消费，大力推广新能源汽车。实施循环发展引领计划，全面推进园区循环化改造、工业绿色化发展、农业循环化发展，支持谷城、老河口打造千亿级再生资源利用产业集群，建成国内一流的餐厨垃圾利用示范城市，创建国家循环经济示范区。实施全民节能行动计划。落实最严格的耕地、林地、湿地保护和水资源管理制度，促进资源节约高效利用。

　　全力推进环境治理。实施水污染防治行动计划，建成100座乡镇污水处理厂，城镇污水排放全部达到一级标准，汉江襄阳段水质稳定在Ⅱ类水平。实施大气污染防治行动计划，力争城市空气优良天数比例达到80%。统筹实施土地污染防治、农村饮水安全、改水改厕、垃圾处理、大型沼气设施建设等工程，农村生活垃圾无害化处理率达到95%，持续改善农村人居环境。

　　全面推进国土绿化。开展“绿满襄阳”提升行动，着力构建市域森林生态系统，五年累计植树造林75万亩，森林覆盖率达到45%以上，形成“城在林中、镇在林中、村在林中、房在林中”的美丽画卷。

　　（四）坚持开放发展，奋力推进双向开放走在全省和汉江流域前列

　　用足用好建设湖北自贸区襄阳片区的新机遇，全力打造湖北融入“一带一路”战略的重要支点。

　　加快建设湖北自贸区襄阳片区。遵循国际贸易和投资通行规则，加强对外交往专业人才队伍建设，打造法治化、国际化、便利化的营商环境。加快建设“四港一区”，构建航空、水运、铁路、公路立体口岸体系，规划建设综合保税区，全力打造国际陆港城市。

　　打造“一带一路”重要节点城市。打通连接亚欧的“铁铁联运”通道，打造汉新欧线上的重要节点；依托铁路物流基地和汉江“黄金水道”，扩大与沿海港口的合作，打通连接亚太的“铁水联运”、“江海联运”通道，融入21世纪海上丝绸之路。

　　加快发展外向型经济。推进对外贸易优化升级，力争每个县（市）培育一个过亿美元的出口产品、一个过亿美元的出口产业、一个过亿美元的出口基地，全市出口过千万美元的工业企业突破50家。更大力度“引进来”、“走出去”，积极扩大国际交流合作，力争集聚襄阳的世界500强企业突破55家，国际友城达到50个。

　　（五）坚持共享发展，奋力推进民生改善走在全省和汉江流域前列

　　牢固树立以人民为中心的发展思想，实施新一轮改善民生行动计划，努力使人民群众的幸福感、安全感、获得感显著增强。

　　确保精准扶贫不落一人。按照精准扶贫“看三代、管长远”的要求，探索建立长效机制，全力推进扶贫攻坚三年行动计划，确保到2017年底4个插花贫困市（区）建档立卡扶贫对象稳定脱贫、贫困村出列，到2018年底3个贫困县建档立卡扶贫对象稳定脱贫、贫困村出列，实现“摘帽”目标。

　　推进社会保障覆盖全民。实施全民参保登记计划，健全社会救助体系和住房保障体系，力争实现应保尽保。实施更加积极有效的就业创业政策措施，实现更加充分更高质量的就业。构建和谐稳定的劳动关系，逐步提高最低工资标准，千方百计增加城乡居民收入。统筹做好社会福利、社会慈善、社会养老、优抚安置、法律援助等工作，支持红十字、残疾人事业发展，保障妇女和未成年人权益。

　　促进基本公共服务人人享有。坚持教育优先发展战略，全面普及15年基础教育，全力推进襄阳教育园区建设，构建具有襄阳特色的职业教育体系。推动文化大发展大繁荣，加强“文化小康”建设，提高国家公共文化服务体系示范区建设水平，促进全民健身和竞技体育全面发展；加强优秀历史文化的保护和利用，充分释放历史文化名城效应；大力培育和弘扬社会主义核心价值观，不断提升公民思想道德素质和科学文化素养，早日跻身全国文明城市行列。深化医药卫生体制改革，健全公共卫生和医疗服务体系，争创国家健康城市，提高全市人均寿命。坚持计划生育基本国策，促进人口均衡发展。

　　营造和谐稳定社会环境。深入推进法治建设，全面开展“七五”普法，深化司法体制和公安改革，提升法治城市建设水平。提高社会治理社会化、法治化、智能化、专业化水平，完善立体化、信息化社会治安防控体系和矛盾纠纷多元化解机制，打造全国全省平安建设示范区，不断加强安全生产和食品药品安全监管，促进人民群众安全感持续增强。

　　三、2017年的主要任务

　　2017年，是落实市十三次党代会部署的起步年，必须认真贯彻落实中央和全省经济工作会议、省委十届八次全体（扩大）会议精神，奋力推进全市经济稳中求进、进中向好，确保社会和谐稳定，以优异成绩迎接党的十九大胜利召开。

　　当前我国经济正面临速度换档节点、结构调整节点、动力转换节点，我们务必牢牢把握大趋势、引领新常态、遵循大逻辑，坚持稳中求进、好中求快，突出问题导向，持续精准发力，推动转型跨越提速提效。面对经济结构性失衡的矛盾，更加注重深化供给侧结构性改革，走生态优先、工农业协调发展之路，着力在补齐产业、民生、基础设施短板上持续突破；面对经济增长内生动力不足的矛盾，更加注重培育新的发展动能，以提高质量和核心竞争力为中心，以扩大有效投资为支撑，推动新兴产业发展壮大、传统产业焕发生机，着力振兴实体经济；面对市场主体经营分化、困难增多的矛盾，更加注重优化营商环境，千方百计破解要素制约，加强预期引导、提振发展信心、激发民资民企活力，着力凝聚全市“提升首位度、打造增长极、争当排头兵”的强大动力。

　　今年全市经济社会发展的主要预期目标是：地区生产总值增长9%，规模以上工业增加值增长9.8%以上，全社会固定资产投资增长16%，社会消费品零售总额增长12.5%，地方公共财政预算收入增长11%，实际外商直接投资增长10%，出口总额增长5.5%，城镇和农村常住居民人均可支配收入与经济增长同步，全面完成精准脱贫、计划生育、安全生产、节能减排、居民消费价格指数等省定目标。

　　围绕实现预期目标，重点抓好八个方面的工作：

　　（一）着力扩大有效投资

　　全力推进精准招商。坚持大员上阵，整合招商力量，打造“狼性”招商团队，聚焦补齐产业短板，以招“大”引“强”、招“新”引“高”为重点，强力推进“一个龙头、六大支柱”产业链招商，着力突破战略性新兴产业招商，积极抓好消费型产业招商。力争全年引进投资过5亿元的项目200个以上，招商实际投资额突破2400亿元，工业项目投资占比达到70%以上。

　　全力推进重点项目建设。扎实抓好136个省市重点项目建设，力争完成投资559.2亿元。扎实抓好400个亿元以上工业项目建设，力争竣工投产200个以上，完成工业投资1800亿元；全力推动猛狮动力电池、际华生态纺织、新火炬汽车电子等67个投资过10亿元的重大工业项目建设，力争东风井关农机、三环车桥精密生产基地等30个项目竣工投产。

　　全力推进重大基础设施建设。大力支持蒙华铁路、郑万高铁、汉十高铁建设，开工建设东津高铁站。加快推进4条高速公路和河谷汉江公路大桥、宜城汉江公路二桥建设，老谷高速建成通车，新建一二级公路150公里、农村公路1000公里。快速推进雅口航电枢纽工程，确保新集枢纽开工建设。完成襄阳机场改扩建工程，组建襄阳航空公司。全力服务和支持鄂北水资源配置工程，抓好华电供热、农网改造等能源项目建设。

　　（二）着力推进工业转型升级

　　推进企业技改提质。大力实施企业技改转型三年行动计划，全年完成技改投资850亿元以上，力争200个重大技改项目竣工投产。实施质量提升行动，培育“工匠精神”，打造“百年老店”，力争重点产品质量抽检合格率高于全省平均水平。深入实施“双百”企业成长工程，开展制造业单项冠军培育提升行动，力争新增规模以上企业120家，新增5家产销过10亿元、15家过亿元的骨干企业。

　　壮大新兴产业规模。大力实施新兴产业倍增计划，力争竣工投产亿元以上新兴产业项目100个，新兴产业产值突破1200亿元。全力推进“中国新能源汽车之都”建设，着力在引进整车项目上实现新突破，力争美洋汽车实现量产，国能动力电池、雅致和东津新能源汽车项目投产，新能源汽车产值突破180亿元。大力支持在襄军工企业发展，积极推进“民参军”，力争军民融合产业产值突破650亿元。

　　培育特色产业集群。创新特色产业园区投融资和运营机制，推动20个特色园区公共服务平台全覆盖、6个新认定园区基础设施全覆盖，提升产业集聚功能。引导、支持6个县（市）和襄州区各培育2-3个优势产业，新开工1个投资过10亿元、2个过5亿元、10个过亿元的优势产业项目，加快形成“一县一品”发展格局。

　　优化企业发展生态。全面落实国家和省降成本政策措施，进一步减轻企业负担。全面落实万亿工业强市建设措施，支持企业做大做强。完善“千名干部进千企”常态化机制，全力帮助企业排忧解难。优化金融生态环境，千方百计化解企业融资难、融资慢，力争实现信贷投放高于全省平均增幅、涉农和中小企业贷款高于全部贷款增幅。

　　（三）着力推进农业供给侧结构性改革

　　大力发展绿色农产品生产。开展绿色高产高效示范，大力推广绿色高产高效种植模式，狠抓农产品标准化生产、品牌创建、质量安全监管，支持保康县建成农产品质量安全追溯系统，适时在全市推广，让老百姓吃得更安全，把襄阳特色优质农产品品牌树起来。加快发展特色农业和林特产业，打造“一镇一业”专业镇，创建“一村一品”示范村。大力推进高标准农田建设，提高粮食综合生产能力。稳定发展生猪和家禽养殖业，加快发展水产业，突破性发展牛羊养殖业，大力推广肉牛高效绿色养殖模式，年出栏肉牛80万头以上，着力打造汉江肉牛产业带。

　　大力培育新型农业经营主体。创建市级以上示范农民专业合作社50家、示范家庭农场50家，培养新型职业农民6300人。大力发展各类农村经营性服务组织，推广“龙头企业+基地+合作社（家庭农场）+农户”的发展模式，加快建立“管理在县、服务在乡”的农村公益性社会化服务新机制。实施信息进村入户工程，提高农业信息化水平。

　　大力推进农村一二三产业融合发展。推动农产品加工业转型升级，着力引进品牌食品、木本油料、中药材和家具加工等行业龙头企业，带动农业全产业链建设。以推进农工融合、农旅融合、农电（商）融合为重点，加快培育一二三产业融合发展新业态。加快建设“中国有机谷”，推动有机食品生产关键技术研究，着力引进有机肥、生物农药、有机食品加工等行业龙头企业，壮大有机产业规模。

　　大力推进农村改革。有序推进农村土地“三权分置”，全面完成农村承包土地经营权确权颁证，发展适度规模经营。积极推进宜城农村土地征收、集体经营性建设用地入市、宅基地制度改革试点，确保通过国家验收。积极推进农村集体产权确权到户和股份合作，继续深化集体林权制度改革，全面完成国有林场改革。全面落实惠农助农政策，积极化解农村债务，排农忧、解农难、安农心、促农稳。

　　（四）着力壮大现代服务业规模

　　突破性发展大数据产业。全面实施大数据行动计划，推动政府数据开放共享，支持华为、中国移动、中国电信打造区域性大数据中心 ，构建全市统一的基于互联网的政务服务、产业发展和信息惠民平台；支持华为、中国电信大数据产业园建设，力争引进行业领先企业5家以上。

　　突破性发展现代商贸流通业。推进专业市场向樊西新区集聚，吸引世界500强商贸物流企业落户。推进重点电商园区产业发展，大力发展农村电子商务，力争电子商务交易额突破1000亿元。推进乡镇和村级商贸物流设施建设，健全农村物流配送网络。加强商贸流通标准化建设，大力发展智慧物流。

　　突破性发展文化旅游业。扎实抓好襄阳古城、古隆中、岘山、唐城等重点旅游项目建设，力争古隆中创5A通过验收，新增2家4A级景区。大力发展乡村旅游，支持县（市、区）开展试点示范，着力打响谷城茶旅小镇、保康风情小镇、南漳古山寨、襄城卧龙古镇、老河口水乡花都等乡村旅游特色品牌。加快建设旅游服务设施，建成智慧旅游云数据中心。

　　突破性发展大健康产业。着力培育健康养老产业，完善城乡养老服务体系，每个县（市、区）举办1-2个医养结合模式试点，积极探索养老机构“公建民营”新机制，鼓励社会力量兴办养老服务机构。着力培育健康养生产业，规划建设业态多样的特色养生小镇。着力培育健康运动产业，规划建设汉江两岸、引丹干渠、鱼梁州环岛等自行车和马拉松赛道，积极争取承办全国性运动赛事。

　　（五）着力推进大众创业万众创新

　　加强科技创新能力建设。大力支持骨干企业与高校、科研院所共建研发平台，鼓励军工科研平台和设备设施开放共享，加快建设检测认证产业园二期工程，规划建设研发设计产业园，积极创建国家制造业创新中心。力争全年实施产学研合作项目80个，获得授权专利2200件，新增高新技术企业30家。

　　提升创业创新载体功能。加快建设襄阳科技城、东津科创孵化中心等“双创”集聚区，完善襄阳市大学科技园、襄阳电商创业谷、襄阳创业大街等“双创”平台功能，支持县（市、区）建设创业园区、梦想小镇。继续办好全国大学生方程式汽车大赛，建成永久性大赛举办地。大力发展以“互联网+N”为基础的众创空间，支持优客工场与东风公司合作共建汽车产业众创空间，力争新增省级以上科技企业孵化器和众创空间5家。

　　壮大创业创新主体。做强创业创新投资平台，设立汉江天使投资基金，建立人才创新创业超市，加大“双创”服务力度。举办高水平的创业大赛，着力打造全国有影响的“双创”活动品牌。力争全年引进企业紧缺急需的高层次创新人才200名以上、高端人才创业创新团队20个，新增大学生创业者3000人以上，新增各类市场主体8.5万户以上。

　　（六）着力推进美丽襄阳建设

　　加快建设宜居城市。扎实抓好609个重点城建项目，力争完成投资650亿元。积极推进东津新区建设，扎实抓好东津高铁站城市综合配套工程，加快建设“两馆两中心”，建成市民中心、文化艺术中心、科技馆。全力推进棚户区改造，力争棚改货币化补偿全覆盖，推动棚改房屋征收提速，重拳打击违法建设，科学规划和建设棚改征迁区,力争完成棚户区改造投资200亿元以上；合理调控房价，促进房地产市场健康平稳发展。实施畅通工程，开工建设南外环线，实施内环线快速化改造，启动东西轴线和南北轴线延伸工程，加快推进庞公大桥、苏岭山大桥和清河一桥拓宽工程，加快形成外环闭合、内环畅通、组团互联的城市快速路网体系。优先发展公共交通，积极创建“公交都市”。完善市政设施功能，积极推进城市综合管廊和海绵城市建设，加快实施城市应急备用水源工程，新建5座排涝泵站。实施城市修补和生态修复工程，启动襄水河等水系连通工程，加快实施汉江景观带、公园绿地、生态湿地等景观项目；建成东津污水处理厂，完成襄水河、大李沟水体治理任务。健全“大城管”体制，完善考评机制，提高精细化管理水平，让城市更加有序起来。

　　加快建设特色小镇。坚持产业建镇，打造经济强镇。深挖、延伸、融合产业功能、文化功能、旅游功能和社区功能，打造让人愿意留下来创业和生活的特色小镇。做好整体规划和建筑风格个性化设计，建设传统与现代、历史与时尚、自然与人文完美结合的特色小镇。坚守生态底线，建设环境优美的特色小镇。创新城镇化发展体制机制，扎实抓好宜城、老河口国家新型城镇化综合试点工作，确保宜城试点通过中期评估；全面实施居住证制度，积极推进“人地挂钩”试点。

　　加快建设美丽家园。大力实施“绿满襄阳”提升行动，建成市级绿色乡村290个、省级绿色示范乡村120个。大力实施“蓝天碧水净土”工程，重拳整治大气污染，确保空气质量持续好转；严格落实汉江流域水环境保护条例，全面推行河长制，完成汉江襄阳段污染源排查整改工作，完成所有工业集聚区集中式污水处理厂建设，确保水生态环境持续改善；实施土壤污染防治计划，推进污染场地修复。大力推进美丽乡村建设，加强农村环境综合整治，建成“村收集、镇转运、县处置”的农村垃圾处理体系，实现乡镇和村医疗废弃物全收集、全处理；统筹规划实施大型沼气工程，采取政府购买服务方式，探索建立社会化、市场化、专业化的运营管理机制。

　　（七）着力深化改革开放

　　深化行政管理体制改革。积极推进“放管服”改革，扎实办好全省相对集中行政许可权改革试点，探索“先批后审”和工业项目“零审批”，全面推行联审联批和网上审批，推行“多证合一”“一照一码”“证照分离”登记制度；构建“大市场监管”格局，全面推行“双随机一公开”监管；推行“网上办公”，建设“线上政府”。继续推进市直重点行业领域综合执法体制改革，开展市属高校、公立医院编制备案试点，做好生产经营类事业单位转企改制工作。

　　深化经济体制改革。创新投融资体制，完善汉江产业基金运营机制，积极对接长江产业基金，每个县（市、区）设立一支产业基金，支持招商引资和项目建设；开展PPP项目推进年行动，推动政府和社会资本合作实现新突破；力争推进企业主板上市取得积极成效，新增新三板挂牌企业15家、四板挂牌企业150家以上；大力引进私募股权基金，积极扩大债务融资。继续深化财政体制和“营改增”等税收体制改革，加大财政资金统筹使用和预决算公开力度。深化国企国资改革，加快改组和组建专业化国有资本投资运营集团，支持地方国有企业多渠道上市，鼓励国有资本参与上市公司增资扩股。

　　推进更深层次更高水平双向开放。科学编制实施湖北自贸区襄阳片区总体规划和方案，加快建立与自贸区发展相适应的行政管理体制机制，加强双语标识、外文网站等公共服务设施建设；完善保税物流中心功能，确保封关后顺利运营，积极开展跨境电商贸易；加快一类航空口岸申报工作，积极推进邮件快件集散中心、国际旅行卫生保健分中心和应对国外技术性贸易措施公共服务平台建设；做好内河港口口岸申报筹备工作，开工建设铁路物流基地、国际陆港货运中心，规划建设小河港出口加工区；加快建立国际贸易“单一窗口”，积极筹建多式联运海关监管中心。加快发展外向型经济，力争在培育外贸主体、引进加工贸易企业、建设出口基地上实现新的突破，支持襄阳轴承、金鹰重工、骆驼股份在“一带一路”沿线国家建设合作产业园。

　　（八）着力增进人民福祉

　　扎实推进精准扶贫。推动精准扶贫、精准脱贫政策措施落地生根，鼓励社会力量积极参与，因村因户因人精准施策，加大产业就业扶贫力度，促进贫困户持续稳定增收。突出抓好南河流域脱贫攻坚，力补基础设施短板，支持村集体经济做大做强，增强“造血”功能。

　　提高就业和社会保障水平。切实加强就业工作，全年城镇新增就业8.2万人、扶持创业2500人，创业带动就业1.1万人。推进城乡居民基本医疗保险和机关事业单位养老保险制度改革；全面开展重特大疾病医疗救助工作，进一步提高社会救助标准。提升自然灾害应急救助能力，力争创建8个国家和省级综合减灾示范社区。

　　加快发展社会事业。大力发展教育事业，继续实施第二个学前教育三年行动计划，加快推进县域城乡义务教育学校一体化改革，进一步扩大优质高中资源，推动职业教育、高等教育加快发展；确保市三中新校区建成投入使用，启动中心城区5所中学迁建改造工程，规划建设湖北文理学院新校区，积极筹建襄阳外国语学校。加快发展公共文化事业，大力推进市县重点文化体育场馆和综合档案馆建设，加强老年大学规范化建设，确保全民健身二期工程竣工，完善基层综合文化中心服务功能；深入开展高水平、群众性、襄阳味的惠民文化体育活动；加强文化遗产保护，扎实推进“万里茶道”申遗工作；大力推进创建全国文明城市工作。积极推进国家健康城市创建，扎实抓好城市公立医院综合改革试点工作，学习推广黄陂健康管理模式，大力发展中医药事业，推广宜城托管乡镇、村（社区）医疗卫生机构的模式，提高计划生育服务管理水平。

　　加强和创新社会治理。把专项治理和系统治理、综合治理、依法治理、源头治理结合起来，深化实有人口、实有房屋和特殊人群服务管理，打造网格化治理新模式；健全立体化、信息化治安防控体系，严密防范、严厉打击各类违法犯罪活动，奋力夺取全国综治“长安杯”。全面加强安全生产和食品药品安全监管，确保国家安全发展示范城市和国家食品安全示范城市创建通过验收。切实加强国防动员，支持国防后备力量建设，抓好新一轮“双拥”模范创建。认真做好民族宗教、对口支援等工作。

　　今年要扎实办好关系民生的十件实事。

　　1、开工建设外环线（绕城高速南段），今年投资8.6亿元，完成汉江特大桥主桥桩基建设，3年内建成通车，实现外环闭合。

　　2、启动、开工建设城区集中供暖工程。淘汰黄标车和燃煤小锅炉，完成加油站油气回收改造和20个工业企业挥发性有机气体治理，实现建设工地扬尘管控全覆盖，空气优良天数比例超过省定目标。

　　3、改造棚户区47021套，其中市区26214套，让更多老百姓早点住上高楼大厦。

　　4、免费为全市农村适龄妇女开展“两癌”（宫颈癌、乳腺癌）筛查，为患“两癌”贫困妇女提供救助。

　　5、新建、改建公办幼儿园35所，增加7000个学位。实施基础教育信息化工程，建成“名校网络课堂”100个、“名师课堂”200个，让全市所有中小学生都能通过互联网享受到优质教育资源。

　　6、完成78个建制镇污水处理厂和垃圾压缩中转站建设，农村生活垃圾收集率达到80%以上；实施100立方米以上沼气工程83个，覆盖农户3000户以上，解决人畜粪便污染问题。

　　7、脱贫10万人，140个贫困村出列。将城市居民最低生活保障标准由每月500元提高到600元，农村居民最低生活保障标准由每月345元提高到400元，农村特困供养人员救助标准提高到9600元左右，社会救助标准进入全省前列。

　　8、中心城区新增绿地500万平方米，建成30公里岘山绿道，启动建设20公里的樊城环形绿道。

　　9、实施农村饮水安全巩固提升工程，解决16万人的饮水不安全问题；全面禁止水库投肥养鱼。

　　10、全年送戏下乡1000场、“欢乐襄阳”文化惠民演出150场，援建农村和社区体育设施300处。

　　四、努力建设人民满意的政府

　　重任千钧惟担当。全市各级政府要切实珍惜人民的信任，始终牢记肩上的使命，以“作风建设年”为契机，着力打造作风硬朗、能打硬仗的公务员队伍，努力建设为民、务实、清廉的人民满意政府。

　　始终做到信念坚定、忠诚履职。“天下至德，莫大于忠”。全市政府系统党员干部必须始终坚持正确的政治方向，严格遵守政治纪律和政治规矩，牢固树立“四个意识”，特别是核心意识和看齐意识，自觉在思想上政治上行动上同以习近平同志为核心的党中央保持高度一致，把绝对忠诚铸入灵魂、融入血脉，铸造坚定的政治品格。坚决维护市委权威，始终把政府工作置于市委的领导之下，不折不扣地落实中央、省委、省政府和市委的决策部署。压实全面从严治党主体责任，加强和规范政府系统党内政治生活，营造风清气正的政治生态，凝聚干在实处、走在前列的强大合力。

　　始终做到敢于担当、奋发竞进。“有多大担当，才能干多大事业”。各级政府和公务人员务必把责任铭记心中，把担当扛在肩上。面对“提升首位度、打造增长极、争当排头兵”、开创“两个中心、四个襄阳”新局面的重大使命，牢固树立进位意识、争先意识、标杆意识，咬定发展不放松，跨越赶超不停步，以舍我其谁的勇气在全省发展大局中争第一、做唯一；面对改革发展的艰巨任务、前进路上的困难挑战、人民群众的重大关切，敢于迎难而上、担难扛责，以敢为人先的精神破障闯关、攻坚克难；面对“本领恐慌”、能力不足的问题，大兴学习之风，建设学习型政府，促进广大公务人员善学善思、善作善成，以履职担当的新能力不断开辟奋发有为、干事创业新境界。

　　始终做到务实重行、苦干实干。施政之要贵在落实，重在实干。各级政府和公务人员务必做到真抓实干、埋头苦干、少说多干。对重点工作制定任务书、路线图、时间表，实行挂图作战、对账销号，精准发力抓落实；健全流程式、全过程督查机制，打造覆盖全市的“互联网+督查”平台，全程紧逼促落实；建立一月一通报、一季一拉练的项目推进机制，促进争先恐后、你追我赶比落实；切实提高执行力和公信力，深入践行“三严三实”，对定下来的事立说立行、马上就办、办就办好，雷厉风行抓落实；强化责任意识，整治为官不为，用责任倒逼落实。各级政府和部门务必发扬钉钉子的精神，一锤接着一锤敲，一步一个脚印地把“两个中心、四个襄阳”的美好蓝图变成现实！

　　始终做到依法行政、从严治政。各级政府及其工作人员务必常怀敬畏法纪之心，牢固树立法治思维，自觉运用法治思维和法治方式推动工作，善于运用地方立法推动改革发展，法定职责必须为，法无授权不可为。扎实做好全省法治政府试点工作，推进依法决策，推行综合执法，切实做到严格规范公正文明执法。自觉接受市人大及其常委会的法律监督和工作监督、市政协的民主监督，加强审计监督和政务公开，充分发挥媒体作用，及时回应社会关切。深入推进政府系统党风廉洁建设，层层签订党风廉洁建设责任书，推动中央八项规定精神全面落实，驰而不息整治“四风”，着力打造廉洁政府。

　　各位代表，我们担负着“提升首位度、打造增长极、争当排头兵”的光荣使命，重任在肩，时不我待，唯有只争朝夕、苦干实干。让我们紧密团结在以习近平同志为核心的党中央周围，在省委、省政府和市委的坚强领导下，不忘初心、继续前进，干在实处、走在前列，为开创“两个中心、四个襄阳”建设新局面，顽强奋斗、艰苦奋斗、不懈奋斗!
